# Acquired Adult Aerodigestive Fistula: Classification and Management

**DOI:** 10.1007/s11605-018-3811-0

**Published:** 2018-06-25

**Authors:** Yassar A. Qureshi, M. Muntzer Mughal, Konstantinos C. Fragkos, David Lawrence, Jeremy George, Borzoueh Mohammadi, Khaled Dawas, Helen Booth

**Affiliations:** 1 Department of Oesophago-Gastric Surgery, University College London Hospital, 250 Euston Road, London, NW1 2BU UK; 2 Department of Medical Statistics, University College London, London, UK; 3 Department of Thoracic Surgery, University College London Hospital, London, UK; 4 Department of Thoracic Medicine, University College London Hospital, London, UK

**Keywords:** Aerodigestive fistula, Tracheo-oesophageal fistula, Oesophageal cancer

## Abstract

**Background:**

Acquired aerodigestive fistulae (ADF) are rare, but associated with a high mortality rate. We present our experience of the diagnosis, management and outcomes of patients with ADFs treated at a tertiary centre. Utilising our findings, we propose an anatomical classification system, demonstrating how specific features of an ADF may determine management.

**Methods:**

A clinical database was searched and 48 patients with an ADF were identified. A classification system was developed based on anatomical location of the ADF and differences in clinico-pathological features based on this categorisation were performed, with the chi-squared test used for inferential analyses and Kaplan-Meier curves with log-rank test to assess survival.

**Results:**

Twenty (41.6%) patients developed an ADF secondary to malignancy, with previous radiotherapy (18.7%), post-operative anastomotic dehiscence and endotherapy (14.6% each) representing other causes. Thirty-one patients were managed with tracheal and/or oesophageal stents and eight underwent surgical repair. The classification system demonstrated benign causes of ADF were either proximally or distally sited, whilst a malignant cause resulted in mid-tracheal fistulae (*p* = 0.001), with the latter associated with poorer survival. ADFs over 20 mm in size were associated with poor survival (*p* = 0.011), as was the use of previous radiotherapy. Proximal and distal ADFs were associated with improved survival (*p* = 0.006), as were those patients managed surgically (*p* = 0.001).

**Conclusion:**

By classifying ADFs, we have demonstrated that anatomical location correlates with the size, history of malignancy, previous radiotherapy and aetiology of ADF, which can affect management. The proposed classification system will aid in formulating multi-modality individualised treatment plans.

## Introduction

Acquired aerodigestive fistulae (ADF) are rare, but confer a high mortality rate. ADFs are largely constituted of tracheo-oesophageal fistulae (TOF). A smaller proportion represent a communication between the oesophagus and the main stem or more distal bronchi or, in patients who have had oesophageal surgery, between the airway and a gastric or colonic conduit.

Up to 50% of ADFs are related to an underlying mediastinal malignancy, most commonly oesophageal (77%), bronchogenic (16%) or thyroid.^1^–^3^ It has been reported that 4.5% of oesophageal malignancies have an associated ADF.^4^ It is uncommon for patients with undiagnosed congenital fistulae to reach adulthood.^5^ Prolonged tracheal intubation—either endotracheal or tracheostomy—is considered to account for up to 75% of non-malignant TOFs.^6^ Endoscopic intervention, such as the use of oesophageal stents, is an escalating cause of ADFs, particularly in patients requiring repeated endotherapy.^7^,^8^ Following oesophageal surgery, it is postulated that up to 4% of patients may develop ADF which are associated with the use of chemo-radiotherapy and complications such as airway injury during radical lymph node dissection or anastomotic leaks.^4^,^7^

The rarity of this condition and the difficulty in diagnosis have likely contributed to a low reported incidence. With improving multi-modality treatment of malignancy and the increasing use of endoscopic therapy, it is likely that the incidence of ADFs too will increase and become a more significant health issue. The centralisation of specialist services is likely to lead to a better understanding of the disease process, treatment and outcomes. Thus, a unified and consistent approach to the management of ADFs is required. A contemporary classification system based on anatomical considerations—thus replicable—can be invaluable in guiding clinical management and assisting in population-based studies of this condition.

In this report, we present our experience of the diagnosis, management and outcomes of patients with ADF at a tertiary centre. We assess the aetiology and anatomy of these fistulae and consider important clinical factors that contribute to long-term survival. Based on these findings, we propose an anatomical classification system of ADFs and suggest a treatment algorithm based on this categorisation.

## Materials and Methods

A clinical database was searched to identify all patients with either a bronchoscopic, endoscopic or contrast radiology-diagnosed ADF, between January 2005 and January 2017 at our institution. Forty-eight patients were diagnosed with an ADF and included in this study. All patients underwent a bronchoscopy and were discussed at a multi-disciplinary meeting to reach consensus on management, barring those patients who presented with an acute airway emergency where decision-making was mandated immediately. Median follow-up, from ADF diagnosis to last clinical engagement or death, was 11.5 months (range 0–63 months; mean 14.8 months).

### Anatomical Classification

**Fig. 1:**
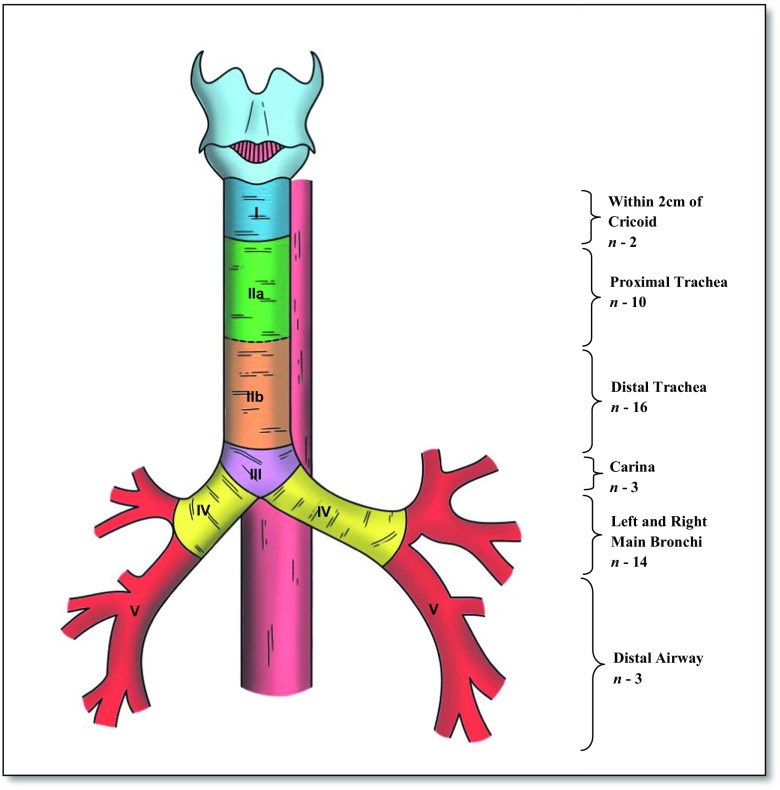
Proposed anatomical classification of aerodigestive fistulae

The measurement of the location and size of the fistulae were determined at index bronchoscopy. Differences in habitus and sex may affect the location when measuring in units of length from a fixed landmark; thus, we standardised our results to present them as a precise position on the airway. Where patients had subsequent bronchoscopy, endoscopy or surgery, the anatomical location and size were corroborated. The airway, in relation to the oesophagus, was stratified into separate anatomical zones, based on reproducibility of diagnosis. Figure 1 highlights this classification system. Using these divisions, the position of the ADF can be clearly delineated at bronchoscopy, as the ADF can be related to clear landmarks (cricoid, carina and main bronchi). Furthermore, these different zones enabled a more structured and relevant analysis of the results, including treatment and survival by location of fistula.

### Statistical Analysis

Continuous data are represented as median and inter-quartile range, and for categorical data, the absolute and relative frequencies are demonstrated. Inferential analyses were performed with chi-square tests. Survival was assessed with Kaplan-Meier curves and log-rank analysis. A *p* value of ≤ 0.05 was considered statistically significant. Survival refers to the time from diagnosis of malignancy or ADF (specified) to the time of last clinical engagement, death or recurrence (confirmed by radiological or pathological investigation). All analyses were performed using IBM SPSS Statistics (release 24.0 2016, Chicago (IL), USA: SPSS, Inc.).

## Results

### Patient Factors and Aetiology of ADF

Table 1 summarises the key patient and disease factors. Twenty-five patients were male and the median age at presentation with fistula was 59 years. Forty-one patients (85.4%) had a current or previous history of malignant disease relating to the oesophagus, airway or head and neck. The median time from diagnosis of underlying disease to the ADF clinically manifesting was 8 months (range 0–144 months). In relation to the aetiology of the ADF, 20 (41.6%) were associated with the presence of an active primary or recurrent tumour (with 21 patients in remission). Other causes included a history of previous radiotherapy related to the site of fistula development (18.7%), previous endotherapy and post-operative anastomotic leak (14.6% each). At the time of index bronchoscopy, the median ADF defect size was 12 mm (range 5–60 mm).

**Table 1:** Key patient and disease factors

	*n*	%
*n*	48	100
Sex		
Male	25	52
Female	23	48
Age (years)	Range 22–69	
	Median 59	
Previous diagnosis		
Malignant disease	41	85.4
Lung/airway	11	23
Oesophageal	26	54.1
Head and neck	4	8.3
Benign	7	14.6
TB	3	6.3
Boerhaave’s syndrome	1	2.1
Treacher-Collins syndrome	1	2.1
Unknown/congenital	2	4.2
Time to fistula development (months)	Range 0–144	
	Median 8	
Primary symptom		
Cough/infection/aspiration	36	75
Respiratory failure/airway obstruction	7	14.6
Dysphagia	4	8.3
Haemoptysis/haematemesis	1	2.1
Aetiology of fistula		
Primary tumour	7	14.6
Recurrent tumour	13	27
Anastomotic leak	7	14.6
Endotherapy	7	14.6
TB	3	6.3
Radiotherapy	9	18.7
Unknown/congenital	2	4.2
History of previous radiotherapy	27	57.4
History of preceding endotherapy	20	42.6
Fistula size (mm)	Range 5–60	
	Median 12 mm	
Management		
Surgical	8	16.6
Non-surgical	40	83.4
Conservative	2	4.2
Oesophageal/tracheal stent	31	64.6
Palliative	7	14.6

### Anatomical Distribution of ADF

**Fig. 2:**
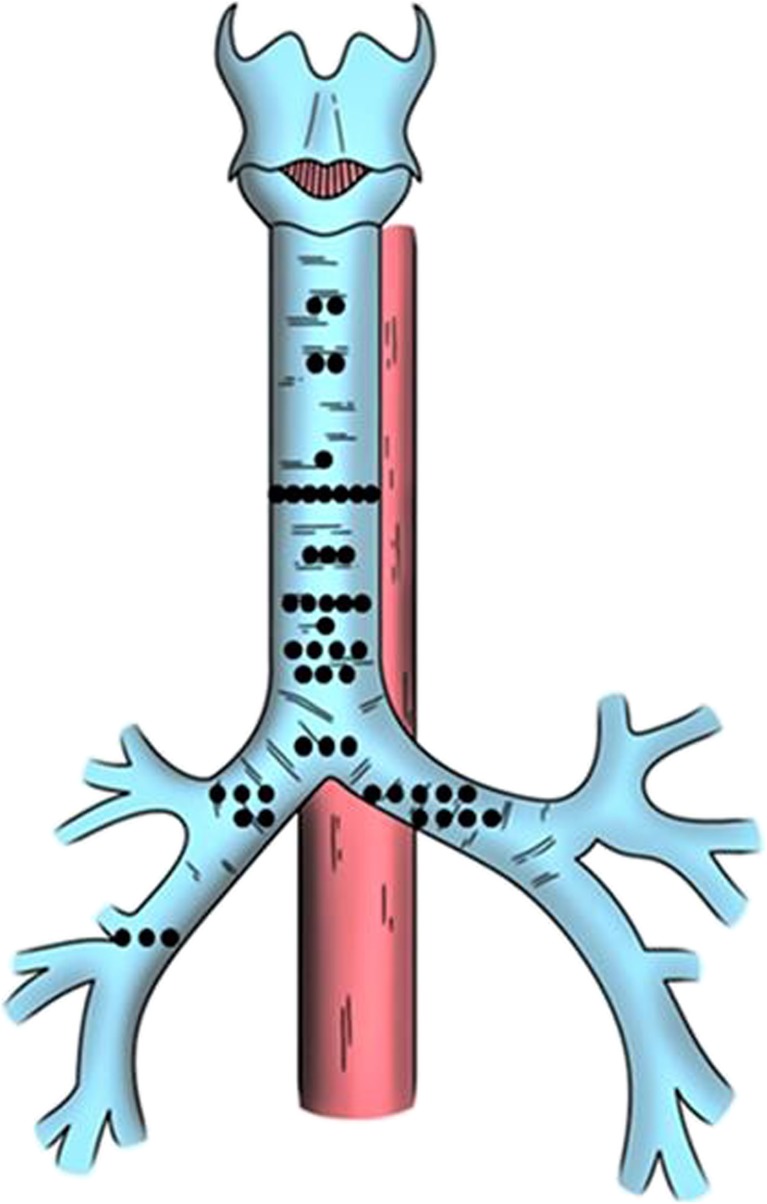
The anatomical distribution of all aerodigestive fistulae (black circle represents each individual ADF in its anatomical position)

Figure 2 highlights the anatomical distribution of all the fistulae. The vast majority of these are TOFs (64.6%; *n* = 31), with fewer affecting the main bronchi (29.2%; *n* = 14) and more distal airway (6.2%; *n* = 3). Based on the classification system (Fig. 1), 2 were proximal (within 2 cm of the cricoid), 10 and 16 affected the proximal and distal body of the trachea respectively, 3 were peri-carinal and 14 within the main bronchi. Three ADF affected the more distal airway or lung parenchyma.

**Fig. 3:**
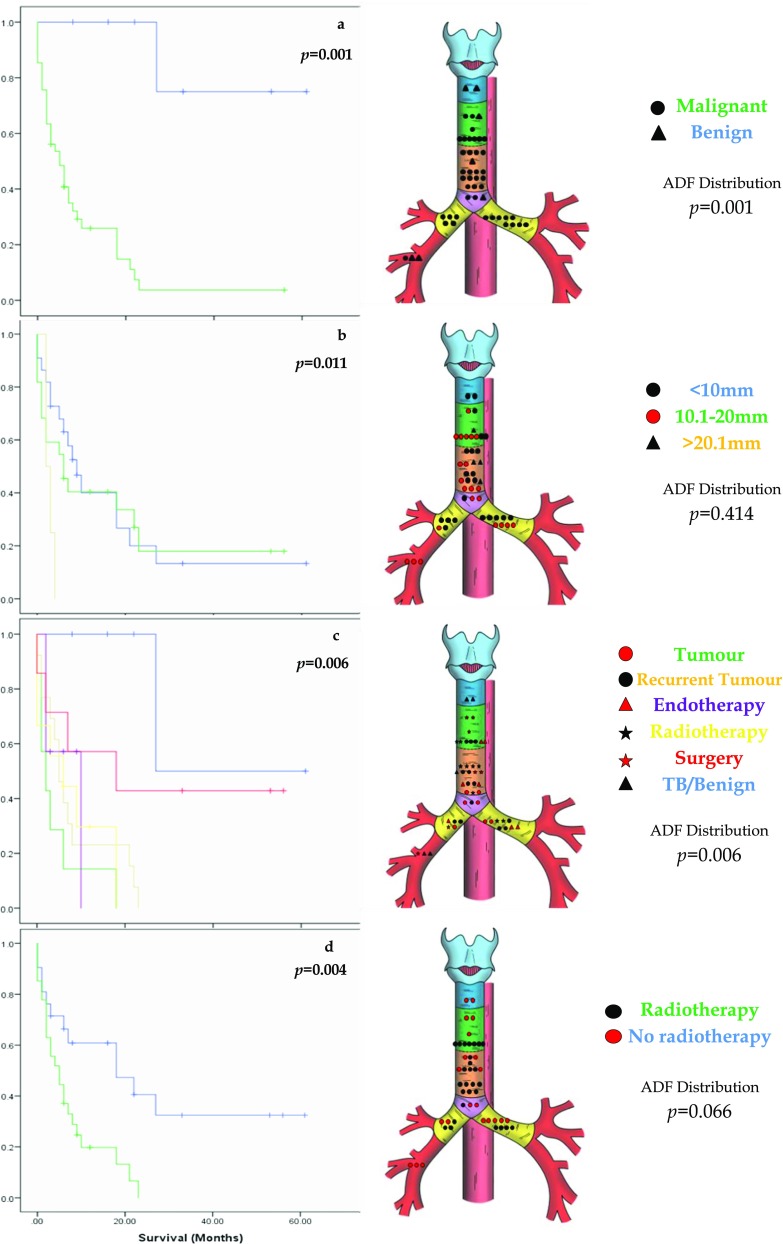
The Kaplan-Meier survival curves (*y*-axis denotes cumulative survival) and ADF anatomical distribution for **a** previous history of malignant disease, **b** size of ADF, **c** aetiology of ADF, and **d** history of previous radiotherapy

There was no difference in the distribution of ADFs based on sex or age at diagnosis. We assessed the original aetiology prior to the diagnosis of an ADF (Fig. 3a): benign disease results in more proximal (type I) and distal (type V) fistulae and malignant disease precedes more centrally placed and main bronchus fistulae (types II and IV). These differences in distribution were significant (*p* = 0.001). However, when organ of tumour origin was assessed for patients with a previous malignant diagnosis, there was no difference in anatomical distribution of ADF (*p* = 0.221). Although there was no statistical significance in distribution of the ADF based on size (*p* = 0.414), all fistulae over 20 mm were type II. Those under 20 mm were more widely distributed across the airway (Fig. 3b).

The underlying cause of ADF was determined to be a primary or recurrent tumour in 41.6% (*n* = 20; Fig. 3c). These were mainly type IIb, III and IV. The cases related to tuberculosis or benign disease were either very distal (type V) or proximal (type I). Where no tumour or benign disease was actively present, we investigated a further potential cause of the fistula and assessed common risk factors for developing an ADF. Seven related to previous surgery, mainly an anastomotic or post-emetic leak. These were mainly type II, save two distal (type V) cases. Previous radiotherapy, with no further identifiable disease process or recurrence, was noted in 9 patients (18.7%). All are type II and IV fistulae, but distribution did not gain significance (Fig. 3d). In total, 27 patients (57.4%) had received radiotherapy, the majority of whom had squamous cell carcinoma of the proximal oesophagus where definitive chemo-radiotherapy is the preferred treatment in the UK.^9^ This regimen comprises 50.4 Gy of radiation, although up to 71 Gy can be delivered.^10^,^11^ However, increasing radiation dosage and number of fractions did not correlate with increased risk of future fistula development. Twenty patients (42.6%) underwent repeated dilations for benign or radiotherapy-related oesophageal strictures or had stents placed and, in the absence of other causative factors, endotherapy was designated the cause of ADF in seven patients (14.6%). The distribution was mainly types II and IV, in keeping with the site of previous disease and treatment (*p* = 0.353).

Overall, the differences between anatomical classification and the cause of fistula demonstrated significance (*p* = 0.006; Fig. 3c).

### Management of ADF

**Fig. 4:**
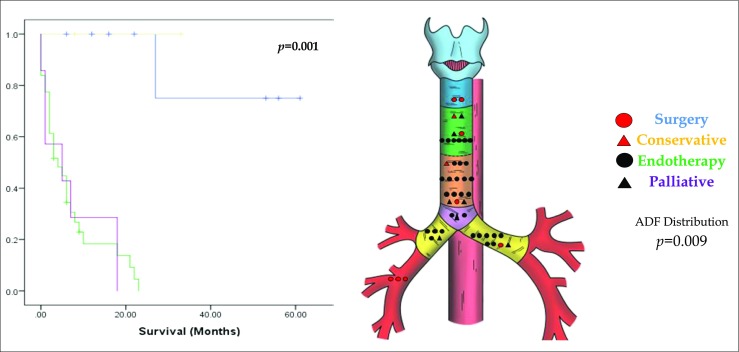
The Kaplan-Meier survival curve (*y*-axis denotes cumulative survival) and anatomical distribution for treatment of ADF

Eight patients underwent surgical repair of the ADF, mainly type I or type V fistulae. Of the cases managed non-surgically, 2 patients were managed expectantly, 7 patients with symptom control (all had locally advanced or distant malignancy), and 31 patients were managed with either a tracheal and/or oesophageal stent (Table 1; Fig. 4).

Of the surgically managed patients, three patients underwent an exploration of the ADF with primary repair of the defect with muscle interposition, either through a neck incision (type I) or thoracotomy. Two patients underwent initial oesophageal exclusion due to heavy local contamination, with subsequent staged reconstruction. Two patients with previous tuberculosis had a lung segmentectomy and lobectomy respectively with concurrent ADF repair, and a further patient in remission after treatment for malignancy underwent oesophageal resection with synchronous reconstruction. This latter patient had an oesophageal stent placed prior to surgery as a temporising measure whilst respiratory and nutritional optimisation was pursued. She had a previous stent placed for a radiotherapy-related stricture, which had eroded into the airway. Thus, it was determined that surgery would provide the optimal treatment strategy for her given previously failed endoscopic therapy and the nature of her oesophageal tissue. Three patients suffered a complication, with one post-operative mortality at 4 months. The remaining seven patients all achieved normal oral alimentation, with no evidence of ADF recurrence, at a median follow-up of 32 months.

Forty patients were managed non-surgically. Of these, two were very small fistulae in the absence of debilitating symptoms, which were managed conservatively. Seven patients presented with acute respiratory compromise secondary to advanced malignancyand were treated palliatively. Thirty-one patients (64.6%), many of whom had a concurrent malignancy, were managed with either an oesophageal or tracheal stent, and some with a dual stent. Most of these were type II fistulae (*n* = 18), but also included 11 patients who had type IV fistulae in the left or right main bronchus, where either an oesophageal or bespoke bronchial stent was placed. There was a statistical difference between anatomical classification and the type of treatment instituted (*p* = 0.009; Fig. 4). There was also a significant difference in survival (*p* = 0.001), with those receiving surgical treatment or a conservative approach demonstrating far improved survival compared to the other groups.

### Survival

There was no difference in survival based on sex or age. Benign rather than malignant disease was significant (*p* = 0.001) for survival (Fig. 3a), and as the Kaplan-Meier curve in Fig. 3c demonstrates, there was improved survival for patients with a benign and post-surgical cause of fistula, with the poorest outcomes in patients with a fistula secondary to an active malignancy (*p* = 0.006). Of note, patients with a prior malignant diagnosis had a median survival of 6 months (range 2–18 months) after fistula treatment, irrespective of cause of fistula. Organ of tumour origin in patients with a previous malignant diagnosis was not significant for survival (*p* = 0.462). However, there was improved survival in patients with an ADF less than 20 mm (*p* = 0.011), compared with those over 20 mm (Fig. 3b). Patients who had previous radiotherapy also suffered worse survival (*p* = 0.004; Fig. 3d). However, a history of previous endotherapy had no effect on survival (*p* = 0.306).

A total of 36 patients (75%) died after the diagnosis of ADF. Twenty-eight (78%) of these were due to the presence of locally advanced, distant or recurrent malignancy, and 8 (22%) due to acute respiratory failure. The median survival in these 36 patients was 6 months (range 0–27). Six patients (12.5%) died within 30 days of diagnosis of ADF and a further four (8.3%) within 60 days. Twelve patients were alive at last clinical follow-up, with no evidence of fistula recurrence, at a median time of 27.5 months (range 13–63).

Patients who underwent surgery (*n* = 8) had better long-term outcomes, with seven patients alive at a median of 32 months (range 4–63). Two patients treated conservatively are disease-free at a median of 25.5 months. Patients who were managed with a stent or in a palliative setting had poor survival, with 35 patients suffering death at a median of 6.5 months from time of diagnosis of ADF (range 1–33). Of this cohort, a further three patients with a diagnosis of oesophageal SCC remain alive at a median of 26 months (range 13–29). All had an ADF managed with stent placement and definitive chemo-radiotherapy.

**Fig. 5:**
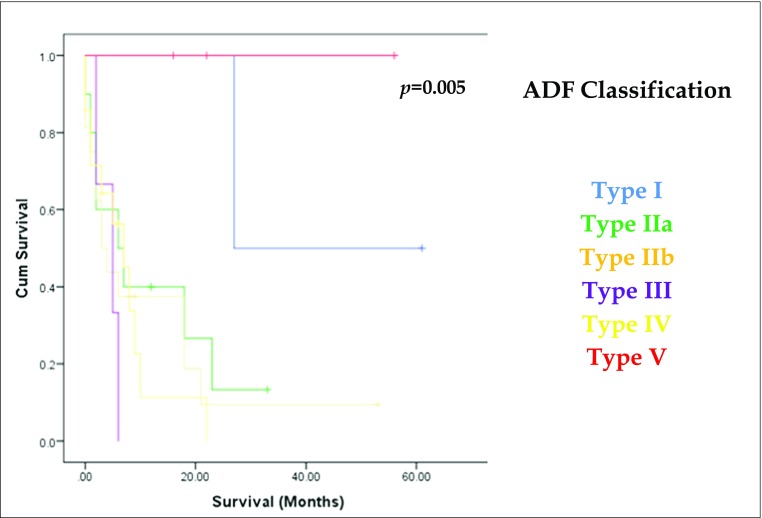
The Kaplan-Meier curve demonstrating survival for each classification of ADF

Figure 5 and Table 2 demonstrate survival based on the classification of the fistula. Type I and type V have superior outcomes, with poorer outcomes for type II, III and IV ADFs. The differences in survival by classification of ADF are significant (*p* = 0.05).

**Table 2:** Median survival by fistula classification

Fistula classification	Median survival (months)	IQR (months)
I	44	27–54
IIa	6	2–23
IIb	3	1–18
III	5	2–6
IV	7	1–9
V	52	36–69

*IQR* interquartile range

## Discussion

Aerodigestive fistulae are associated with high morbidity and poor survival. Paucity of literature makes the true incidence of ADFs difficult to establish, but several factors may explain the apparent low incidence. ADFs can present acutely, where the diagnosis of a fistula is not established before death ensues. In others, an underlying diagnosis of malignancy is sufficient to explain symptoms, and thus, specific investigations to assess for a fistula are not pursued. Indeed, many patients with mediastinal malignancy present with metastatic disease, where palliative care is the only option. Those with long-term survival after malignancy who develop ADFs often have a spurious diagnosis of chronic obstructive airway disease, explaining the recurrent chest infections. Most cases are detected either by careful clinical evaluation with a low index of suspicion or incidentally during the course of respiratory assessment or oncological surveillance. With better reporting, improving diagnostics and longer cancer survivorship, the incidence of ADFs is likely to rise, thus making a consistent approach to all facets of its clinical care an important healthcare consideration.

We developed a classification system based on the anatomy of the airway. Most previous references to ADFs are based on distance of the lesion from a measurable landmark, commonly the incisors. However, such a measurement does not account for physical differences that are inherent in patients, and thus, reproducibility and a wider applicability are lost. By developing an anatomical system, we intend that precise documentation of the location of the ADF is achieved, as this does confer a role in determining management. Landmarks that are easily identifiable at bronchoscopy have been used to develop this classification system: the cricoid, the carina and main bronchi. This facilitates an accurate position to be recognised whilst improving inter-observer replication. Within the context of centralisation of specialist services, it is necessary to have such a system for communicating findings consistently, and also in order to develop a long-term structured approach to measuring, reporting and managing ADF, particularly given the multi-disciplinary nature of care in contemporary practice. Furthermore, concentration of care in specialist centres will enhance the understanding of this condition resulting in improved management and better outcomes. It is for these reasons that a universal classification system is necessary.

There are patterns of airway distribution of ADF based on several factors; an exploration of these can aid in formulating treatment plans. It is clearly evident that the presence of mediastinal malignancy is the greatest determinant of ADF management. Findings such as location, size of defect and previous radio- or endotherapy can be considered secondary factors, which may aid in formulating an *individualised treatment strategy*. Within the malignant ADF group, further risk factors are consequently amplified. For example, the use of radiotherapy and surgery can affect local tissue quality, and endotherapy is often necessary for complications of initial treatment (such as stricture formation requiring repeated dilation or stent placement). A history of previous radiotherapy facilitates the development of ADF owing to the architectural changes that irradiated tissue undergoes. With increasing use of radiotherapy in contemporary practice, and higher radiation doses for definitive therapy, the risk incidence of ADF may increase.^12^ Similarly, the use of oesophageal stents is now commonplace, with an associated risk of airway fistulation.

A multi-disciplinary approach is necessary to determine optimal treatment for each patient. Our classification system aims to enable a comprehensive care plan, unifying treatment of the underlying aetiology as well of the fistula. This will ensure that some patients are not denied standard oncological treatment on the basis of the presence of an ADF, an approach for which there is increasing evidence in the literature.^12^–^14^ In the absence of distant disease, the presence of a fistula indicates locally advanced disease, often beyond the scope of curative therapy. However, most contemporary reports do not consider the presence of an ADF a contraindication to commencing oncological therapy. Optimal strategy here involves the use of a tracheal or oesophageal stent, in order to minimise airway contamination, coupled with the use of radiotherapy, with or without chemotherapy. This strategy has been shown to improve survival.^12^,^13^ In some cases, by treating the tumour, closure of the ADF can be achieved.^14^ Muto et al. achieved a TOF closure rate of 70.8% utilising chemoradiotherapy.^15^ Endoscopic intervention has developed dramatically over the last decade, and the use of stents in particular plays a key role in ADF management. In some cases, they provide excellent palliation, allowing oral alimentation.^16^ In other patients, they provide a temporising measure whilst oncological treatment is instituted. Although a variety of stents are available, most authors favour self-expanding covered metal stents (SEMS) for the management of malignant TOFs, which appear to have improved efficacy and are associated with fewer complications than plastic stents.^17^ The placement of proximal oesophageal stents is precarious, owing to the presence of the cricoid and pharyngeal constrictors, and these often fail. In these instances, a tracheal stent should be placed synchronously, thus providing a “double closure” of the fistula, whilst minimising the risk of subsequent dyspnoea.^18^,^19^ Similarly, ADFs around the carina can be difficult to manage; thus, a dual stent strategy is often employed. Endobronchial stents can be utilised to address main bronchi fistulae. In our practice, unless anatomy precludes, an oesophageal stent is placed in preference to a tracheal stent in the first instance, as these are technically easier to deploy and the procedure is better tolerated by patients. However, it has been noted in several studies that stents can *cause* ADFs, by oesophageal stent erosion into the airway. Repeated dilation or laser use also appears to increase the risk, likely due to small tears in the oesophagus with inadequate healing enabling microscopic changes necessary for ADF development.^7^,^20^ In this series, the previous use of endotherapy did not attain significance, but it likely has a more significant impact in patients who have had a previously treated or severely diseased oesophagus or trachea. In one study, 36.4% of patients who had previous chemo-radiotherapy suffered a life-threatening complication after stent use, compared to 2.5% of patients who had not.^21^ Furthermore, if future surgical intervention is required, the presence of a stent can make this more technically challenging. Patients who develop an ADF soon after an anastomotic leak are difficult to manage, given that airway contamination may be severe and their physiological state compromised. In the first instance, respiratory lavage (bronchoscopic or thoracoscopic) is necessary, coupled with the placement of an oesophageal stent to minimise ongoing contamination. Smaller leaks may heal with conservative management, but complete anastomotic dehiscence requires a cervical oesophagostomy, with reconstruction and repair of the ADF deferred until the patient has improved. With increasing use of energy devices in surgery, inadvertent and unnoticed injury to the airway during thoracic dissection is likely associated with an increased risk of ADF development, particularly in the context of a subsequent leak.^3^,^6^

Surgery offers the best chance of long-term cure of an ADF, with the intention to arrest contamination of the airway whilst enabling normal oral alimentation. However, patients often present in a poor physiological state; thus, aggressive optimisation is vital. If necessary, an oesophageal or airway stent can be placed as a temporising measure to arrest ongoing respiratory contamination, although for these patients, surgery should be considered definitive therapy. As many patients will have had a previous diagnosis of malignancy, search for recurrent disease must be meticulous, as survival in this group is invariably guarded. Surgery is challenging, requiring a high level of expertise, skill and the propensity to work within a multi-disciplinary team. Although details of operative interventions are beyond the scope of this report, several techniques are available to the surgeon. Type I, IIa and some IIb ADFs can be approached via the neck, but the remainder will require a thoracotomy. Secondary factors such as the size of the defect, the nature of previous interventions and the tissue state of the airway and oesophagus become important considerations in determining the *exact* operative intervention. The options available include exploration of the fistula with primary repair of the trachea and oesophagus, using an interposed intercostal or strap muscle flap.^7^,^22^ More radical techniques include oesophageal exclusion, with reconstruction either at the index operation or as a staged procedure.^12^,^23^ Perhaps the most challenging surgery, and one which carries the greatest risk, is oesophago-tracheal resection and reconstruction. This is usually reserved for larger or circumferential fistulae, or where previous intervention has failed.^22^

**Fig. 6:**
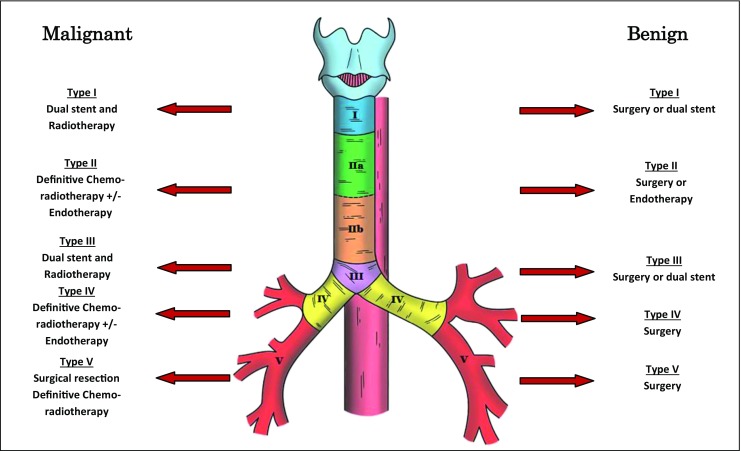
Treatment algorithm for managing ADFs based on anatomical classification. *Dual stent refers to a synchronous tracheal and oesophageal stent

Figure 6 demonstrates a treatment algorithm based on our own experience and on a current evidence-based model, facilitating individualised treatment based on the anatomical location of the ADF. All patients should undergo a bronchoscopy to assess the fistula and the state of the airway, with oesophageal endoscopy performed if further assessment, tissue for histopathology or oesophageal intervention is required. Given the importance of active malignancy in determining ADF treatment, this is considered distinct to benign disease. After this differentiation, secondary factors such as size and state of local tissue should be considered when tailoring *specific* intervention. For benign disease, surgery should form the mainstay of definitive treatment. This is especially true for type I and III ADFs, where the placement of a stent may be technically challenging and likely to fail. For these, if surgery is absolutely contraindicated, a dual stent strategy should be employed. The nature of surgery is dependent on location and secondary factors: a neck incision for proximal ADFs is less morbid than a thoracotomy; for distal and parenchymal fistulae, an en bloc excision of ADF and affected tissue is recommended (thus reducing the risk of repair dehiscence); a staged approach should be strongly considered as a temporising measure if heavy contamination precludes synchronous repair or reconstruction. This latter strategy is especially important to consider in patients who require prolonged optimisation, as exclusion of the ADF along with placement of adequate drains and an enteral feeding tube will facilitate this process. For malignant disease, in the absence of distant metastases, long-term survival and alleviation of ADF-related symptoms should be considered the target. Thus, ADF treatment should be unified with optimal oncological therapy. For type I and III ADFs, a dual stent strategy should be used, to increase the chances of ADF closure whilst radiotherapy is commenced. Similarly, for type II and IV ADFs, stents should be used a temporising measure to curtail ongoing airway contamination whilst oncological treatment is instituted. In the event of a good response, surgery should be re-considered as a secondary intervention if the malignancy is deemed resectable (there is no benefit in debulking surgery, save perhaps for a thyroid malignancy). For type V and some type I ADFs, unless the defects are very small, we advocate surgical resection of the ADF en bloc with the tumour. Although unlikely to significantly affect disease-free survival, it will at least palliate patients from often debilitating symptoms and mitigate the use of stents in problematic positions. Indeed, some patients may subsequently respond well to systemic therapy and become candidates for more aggressive intervention.

### Limitations

ADFs are uncommon and likely underreported. As a result, any study of this disease will involve a relatively small population size. Indeed, by characterising ADFs further, the sample size does become smaller. The development of this classification system was carefully considered in view of the sample size, and the results represent a balance between meaningful analyses and a necessity to divide ADFs based on anatomy and associated therapy. However, given the small sample size, predictive and regression analyses would be underpowered. Similarly, independent predictors of survival are difficult to generate in this study: for such a multi-factorial disease, it would be necessary to involve a much larger study group. There is an inherent selection bias, as patients undergoing surgery all had benign disease and fared better, compared to those with active malignancy. However, we present metrics on these patients and those in remission, who had other causative factors of ADF development, highlighting challenges in management for this group. Furthermore, our experience has led us to develop distinct treatment strategies for those with a benign and malignant cause for ADF (Fig. 6).

### Conclusion

For patients with suggestive symptoms, in conjunction with cognisance of the aetiological risk factors described, a low threshold for investigation into the presence of a fistula is recommended. A reproducible anatomical classification system is invaluable in standardising diagnosis, management and surveillance. We advocate surgical treatment for all benign ADFs. Malignant ADFs should undergo oncological treatment with oesophageal stent placement (with or without tracheal stent). However, although smaller ADFs may close spontaneously, surgery should be considered for larger defects and those who respond favourably to systemic therapy. En bloc resection should be considered for very proximal and distal malignant ADFs, owing to the challenges associated with stent placement in these locations.
